# P-1809. Trends in vaccination and diagnostic testing for SARS-CoV-2 and influenza across the United States, 2019 – 2024

**DOI:** 10.1093/ofid/ofaf695.1978

**Published:** 2026-01-11

**Authors:** Min Kyung Lee, David Alfego, Laura Gillim, Charles M Walworth, Suzanne Dale, Kathryn Lang, Ruth Carrico, Colm Smart, Payman Ghasemi

**Affiliations:** Labcorp, Durham, NC; Labcorp, Durham, NC; Labcorp, Durham, NC; Monogram Biosciences/LabCorp, Laguna Beach, CA; Labcorp, Durham, NC; VaxCare, San Marcos, CA; Norton Healthcare, Louisville, Kentucky; VaxCare LLC, Hooksett, New Hampshire; VaxCare, San Marcos, CA

## Abstract

**Background:**

To assess trends in SARS-CoV-2 and influenza virus testing and positivity rates in populations administered seasonal vaccines.

**Figure:**
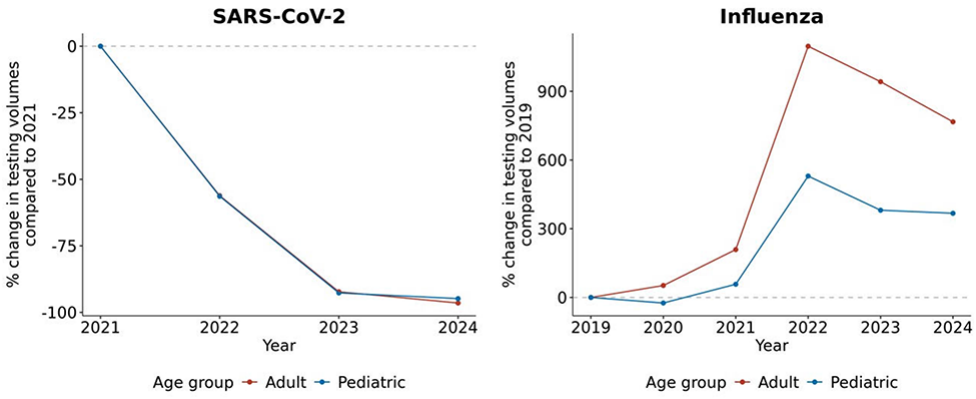
Change in yearly testing volumes for SARS-CoV-2 and influenza in adult and pediatric populations

**Figure:**
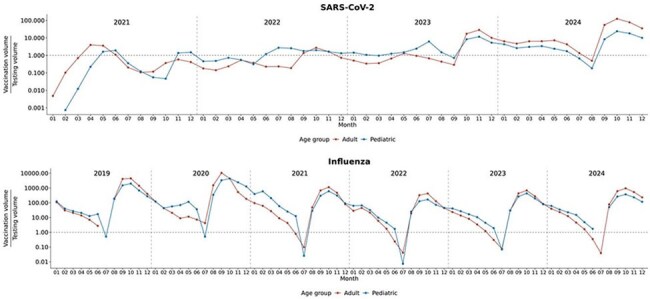
Monthly vaccination to testing volumes of SARS-CoV-2 and influenza in adult and pediatric populations

**Methods:**

We retrospectively reviewed 604,977 vaccination events and 881,573 tests for SARS-CoV-2 and 4,958,425 vaccination events and 31,274 tests for influenza from 2019 to 2024 in a US population administered seasonal vaccines with respiratory diagnostic testing performed at Labcorp. Monthly vaccination volume to testing volume ratio (mVTR) was used to estimate integration of vaccinations and diagnostic testing in health service systems.

**Figure:**
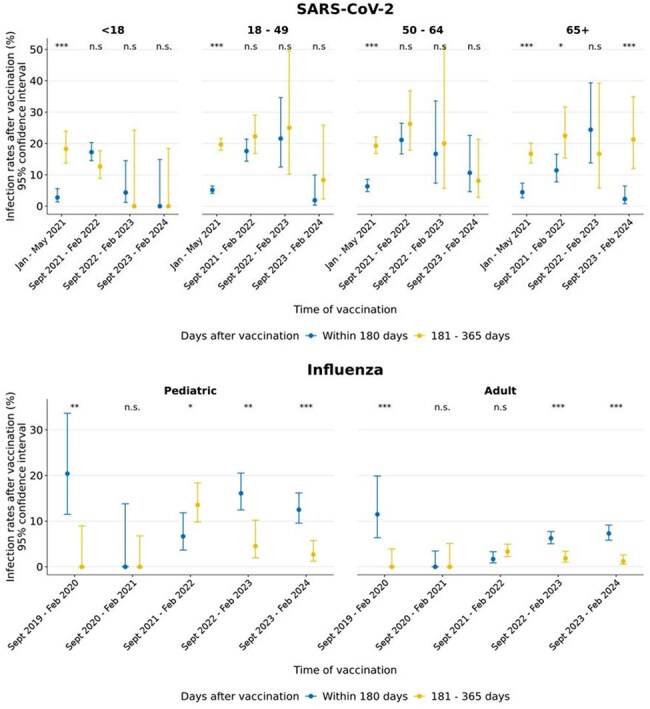
Breakthrough infection rates for SARS-CoV-2 and influenza by age Comparisons of breakthrough infection rates between <180 days and 181 – 365 days performed with two-sample proportion Z-tests. ***: p < 0.001, **: p < 0.01, *: p < 0.05, n.s.: not significant.

**Results:**

SARS-CoV-2 testing decreased 94.8% in pediatrics and 96.5% in adults in 2024 compared to 2021. Influenza testing increased 367.5% in pediatrics and 766.8% in adults in 2024 compared to 2019 (Fig 1). SARS-CoV-2 testing was utilized more than vaccines in adults with mean mVTR = 0.8, SD = 1.0 and was used at similar levels to vaccines in pediatrics with mean mVTR = 1.0, SD = 0.8 during the COVID-19 pandemic (01/2021 – 05/2023) (Fig 2). More vaccines were utilized relative to testing in adults (2024 peak mVTR = 123.4) and pediatrics (2024 peak mVTR = 23.9) after the pandemic. Seasonal variations in SARS-CoV-2 mVTR resembling those of influenza were noted. There were higher volumes of influenza vaccines relative to influenza testing in both adults (2024 peak mVTR = 948.3) and pediatrics (2024 peak mVTR = 370.9).

Breakthrough SARS-CoV-2 infection rates at < 180 days and 181 – 365 days were not significantly different for most vaccination seasons (Fig 3). However, in adults aged 65+ who were vaccinated between 09/2023 – 02/2024 a significant increase in breakthrough infection rates at 181 – 365 days (21.3%) was seen compared to < 180 days (2.3%; p < 0.001). Breakthrough SARS-CoV-2 infection rates were mostly lower with vaccines received 09/2023 – 02/2024 compared to previous vaccination seasons. Breakthrough influenza infections occurred at higher rates within 180 days of vaccination especially in pediatrics.

**Conclusion:**

Interventions for SARS-CoV-2 have recently shifted towards increased utilization of vaccination over diagnostic testing similar to seasonal patterns seen with influenza. Detection of breakthrough SARS-CoV-2 infection may have been impacted by decreased testing in recent months.

**Disclosures:**

Min Kyung Lee, PhD, Labcorp: Employee|Labcorp: Stocks/Bonds (Public Company) David Alfego, PhD, Labcorp: Employee|Labcorp: Employee|Labcorp: Stocks/Bonds (Public Company) Laura Gillim, PhD, Labcorp: Stocks/Bonds (Public Company) Charles M. Walworth, MD, Labcorp: Employee|Labcorp: Stocks/Bonds (Public Company)|Labcorp: Stocks/Bonds (Public Company) Suzanne Dale, PhD, Labcorp: Stocks/Bonds (Public Company) Kathryn Lang, MD, PhD, VaxCare: Advisor/Consultant Ruth Carrico, PhD, DNP, APRN, VaxCare: Advisor/Consultant Colm Smart, MBA, VaXcare: Stocks/Bonds (Private Company) Payman Ghasemi, PhD, VaxCare: Grant/Research Support

